# An Extended Prognostic Index of the ISSWM Score Based on Thyroid Complications in Waldenström Macroglobulinemia/Lymphoplasmacytoid Lymphoma

**DOI:** 10.3389/fonc.2022.870258

**Published:** 2022-05-13

**Authors:** Xinting Hu, Hua Wang, Dai Yuan, Huiting Qu, Ying Li, Na Wang, Xianghua Wang, Xin Liu, Hongzhi Xu, Ya Zhang, Xin Wang

**Affiliations:** ^1^ Department of Hematology, Shandong Provincial Hospital, Cheeloo College of Medicine, Shandong University, Jinan, China; ^2^ Department of Hematology, Shandong Provincial Hospital Affiliated to Shandong First Medical University, Jinan, China; ^3^ School of Medicine, Shandong University, Jinan, China; ^4^ Shandong Provincial Engineering Research Center of Lymphoma, Jinan, China; ^5^ Branch of National Clinical Research Center for Hematologic Diseases, Jinan, China; ^6^ National Clinical Research Center for Hematologic Diseases, The First Affiliated Hospital of Soochow University, Suzhou, China

**Keywords:** Waldenström macroglobulinemia/lymphoplasmacytoid lymphoma, thyroid complications, survival, immunoglobulin M, retrospective

## Abstract

Waldenström macroglobulinemia/lymphoplasmacytoid lymphoma (WM/LPL) is a rare lymphoproliferative neoplasm characterized by clonally related lymphocytes, lymphoplasmacytic cells, and plasma cell proliferation. WM/LPL patients commonly present with elevated immunoglobulin, predominantly immunoglobulin M (IgM). Previous studies reported that thyroid dysfunction was associated with the development and progression of solid tumors. However, only limited information is available on the correlation between thyroid complications and lymphoid malignancies. The aim of our study was to explore the prognostic significance of thyroid complications in WM/LPL. Herein, 13.3% of WM/LPL patients were diagnosed with thyroid complications, which were significantly associated with unfavorable progression-free survival (PFS), overall survival (OS), and adverse treatment response. Co-existing thyroid disease was significantly related to alleviated serum IgM levels, providing an answer to practical problems. Furthermore, the presence of thyroid complications was identified as an independent prognostic indicator for PFS in WM/LPL. Incorporating the ISSWM score with thyroid complications was superior to ISSWM alone in risk stratification and prognostic prediction. Furthermore, subgroup analyses of WM/LPL patients revealed that subclinical hypothyroidism predicted undesirable outcomes at the early stage. These results were also supported by independent microarray dataset analyses. In conclusion, the primary strength of this study is that it provides robust real-world evidence on the prognostic role of thyroid complications, highlighting further clinical concerns in the management of WM/LPL patients.

## Introduction

Lymphoplasmacytic lymphoma (LPL), a rare lymphoproliferative neoplasm with an annual incidence of 3 to 4 cases per million people, is composed of small B lymphocytes, plasmacytoid lymphocytes, and plasma cells that typically involve the bone marrow, lymph nodes, and spleen (1–3). Waldenström macroglobulinemia (WM) is the most common type of LPL, secreting immunoglobulin M (IgM), while IgA and IgG and non-secretory LPL accounted for less than 5%. The clinical presentation of most WM/LPL patients can be attributed to tumor infiltration and/or monoclonal protein, which usually includes disseminated disease, but extranidal involvement and leukemic phase are rare (4–6). According to whole-genome sequencing, MYD88 L265P is the most commonly recurring mutation in the majority of WM/LPL cases (7, 8). Except for MYD88, CXCR4 mutation accounted for 30%–40% of WM/LPL patients, which is the second most common gene mutation (9, 10). The current median survival of WM/LPL patients is approximately 7.4 years [95% confidence interval (CI), 6.8–8.2]. With the advent of novel treatments, survivorship from WM/LPL continues to increase (11–13). When treatment is warranted, combinations of anti-CD20 (rituximab) with alkylating agents and proteasome inhibitors are the principal treatment options. Promising novel therapies in development include non-covalent BTK inhibitors, CXCR4 antagonists, bi-specific antibodies, radioimmunoconjugates, and CD19- or CD20-Targeted Chimeric Antigen Receptor T cells (14–16). Curative therapy is not available and current standard treatment regimens seldom prolong the survival of WM/LPL patients. Therefore, the establishment of prognostic factors is the key to evaluating clinical interventions in WM/LPL (17).

Excessive secretion of monoclonal IgM is the most prominent feature in WM patients (18–20). Although asymptomatic patients do not need to be treated according to current clinical guidelines, those with elevated serum IgM levels are often treated (21–23), because the high level of IgM in the bloodstream increases the risk of hyperviscosity syndrome, which is a potentially life-threatening complication (24–26). In order to evaluate the prognostic significance of IgM levels in a broad range of WM/LPL populations, a survival analysis of serum IgM levels has been carried out in this study.

Data collected by the National Cancer Institute from a large cohort of WM/LPL patients suggest that WM/LPL was correlated with autoimmune disease. Relevant studies have shown that the thyroid is more susceptible to autoimmunity than other organs; thus, thyroid complications have been considered to be the manifestation of autoimmunity problems (27). Furthermore, thyroid function parameters are significantly related to the prognosis of many tumors (28–31). Thus, in addition to hypertension, coronary heart disease, diabetes, and other common complications, our research focused on a major proportion of WM/LPL patients with thyroid complications. To our knowledge, little previous literature have focused on WM/LPL patients with thyroid complications. The aim of our study is to investigate the prognostic value of thyroid complications in WM/LPL.

## Methods

### Study Population

This retrospective observational study enrolled one hundred and fourteen patients with newly diagnosed WM/LPL between May 2001 and March 2021 from Shandong Provincial Hospital with informed consent; no personal identifiable information was recorded in this study. Diagnostic criteria for LPL in all cases were defined as a tumor of small lymphocytes showing evidence of plasmacytoid/plasma cell differentiation without any of the clinical, morphological, or immunophenotypic features of other lymphoproliferative disorders (18). The presence of IgM monoclonal protein associated with ≥10% clonal lymphoplasmacytic cells in bone marrow confirms the diagnosis of WM (32). Baseline demographic and clinical data concerning sex, age, and the International Staging System Waldenström Macroglobulinemia (ISSWM) score were collected from medical records and hospital registries at diagnosis. Eight patients were excluded due to lack of clinical information and laboratory examinations and death with no relation to WM/LPL. All procedures involving human participants in this study were approved by the Research Ethics Committee of the Shandong Provincial Hospital.

### Study Design and Data

In this study, the thyroid complication group was divided according to the clinical diagnosis, from the hospital discharge registry system and electronic medical records (33). There are 13 cases enrolled in this group, including 8 cases with hypothyroidism, 2 cases with Graves’ disease, 1 case with hyperthyroidism, 1 case with thyroid cancer, and 1 case with Hashimoto’s disease but without abnormal thyroid function index. We analyzed 106 WM/LPL patients and collected parameters at diagnosis, including age at diagnosis, hemoglobin level, platelet count, serum albumin, β2-microglobulin, serum lactate dehydrogenase levels, free triiodothyronine (FT3), free tetraiodothyronine (FT), thyroid-stimulating hormone (TSH), anti-thyroglobulin antibodies (TG-Ab), antithyroid peroxidase autoantibody (anti-TPO), and thyrotropin receptor antibody. Subsequently, the survival difference between patients with and without thyroid complications was analyzed in WM/LPL patients. Based on ISSWM, an extended prediction model was established. A subsequent comparison of ISSWM and novel established index was performed. Finally, the role of thyroid-related genes in WM/LPL patients was further investigated.

### Outcomes

The outcome of this study was disease progression or death. Disease progression was defined as ≥25% increase in serum IgM level from lowest nadir and/or progression in clinical features attributable to the disease according to IWWM-7 consensus (34). Patients who died of accidents or other medical conditions with no relation to WM/LPL were excluded from this study. Progression-free survival (PFS) referred to the time from the beginning of treatment to disease progression or death for any cause. Overall survival (OS) was measured from the date of diagnosis to the date of death, which was censored at the date of the last follow-up visit.

### *In Silico* Analyses

The dataset (GSE6691) is from the GEO database (https://www.ncbi.nlm.nih.gov/geo/), and the download data format is MINIML. The Limma package (version: 3.40.2) of the R software was used to study the differential expression of mRNAs. The adjusted *p*-value was analyzed to correct for false-positive results in GEO datasets. To further confirm the underlying function of potential targets, the data were analyzed by functional enrichment. Gene Ontology (GO) is a widely used tool for annotating genes with functions, especially molecular function (MF), biological pathways (BP), and cellular components (CC). To better understand the carcinogenesis of mRNA, the ClusterProfiler package (version: 3.18.0) in R was employed to analyze the GO function of potential targets. The box plot is implemented by the R software package ggplot2; the heat map is displayed by the R software package pheatmap.

### Statistical Analyses

The Chi-square test and Fisher’s precise test were used for the comparison of characteristics. The Kaplan–Meier method was used for the analysis of survival outcomes and the log-rank test was used for comparisons. Cox regression hazard analysis was also performed for univariate and multivariate analysis of OS and PFS. Receiver operating characteristic (ROC) curves and the Delong test were employed for the comparison of two different prognostic scoring systems. *p* < 0.05 was considered significant. All analyses were performed using SPSS software version 26.0 and Medcalc software.

## Results

### Characteristics of WM/LPL Patients at Diagnosis

The study population consisted of 114 WM/LPL patients, with 8 patients excluded because of inaccurate diagnosis and incomplete clinical information. The median age of the remaining 106 patients was 63 years (range, 14 to 86) at diagnosis, and patients over 65 years of age accounted for 41.5% of all patients. The frequency of B-symptoms was 29.2%, and 21.7% of patients showed involved extramedullary sites. More than half of the patients (63%) had a hemoglobin level lower than 11.5 g/dl, while only 21.7% of patients had thrombocytopenia. Almost all patients had an elevated IgM level, which ranged from 0.27 g/L to 126 g/L (median, 23.85 g/L). Among all patients, the most common complication was hypertension (18%), followed by diabetes mellitus (15%) and thyroid function abnormalities (12.3%). The positive MYD88-L265P fusion gene accounted for 73.5% of WM/LPL patients. A rituximab (anti-CD20 antibody)-based combination regimen was the first choice in 41.7% of WM/LPL patients, followed by a Bortezomib (proteasome inhibitor) combination regimen in 10.7%. The characteristics of the study population are summarized in **Table 1**.

**Table 1:** Clinical and biological characteristics of Waldenström macroglobulinemia/lymphoplasmacytoid lymphoma (WM/LPL) patients.

Characteristics		*N*	%
Gender	Male	72	67.9
	Female	34	32.1
Age (median, 63 years; range, 14–86 years)	≤65	62	58.5
	>65	44	41.5
B-symptoms	No	75	70.8
	Yes	31	29.2
Serum LDH level	Normal	51	82.3
	Elevated	11	17.7
Hemoglobin level	≤11.5 g/dl	58	63.0
	>11.5 g/dl	34	37.0
Platelet	≤100×10^9^/L	20	21.7
	>100×10^9^/L	72	78.3
β2-MG	≤3 mg/L	34	39.5
	>3 mg/L	52	60.5
Serum IgM	≤70 g/L	70	85.4
	>70 g/L	12	14.6
IPSSWM risk group	Low (0–1)	27	37.0
	Intermediate (2)	18	24.7
	High (3–5)	28	38.3
Extramedullary involvement	No	83	78.3
	Yes	23	21.7
MYD88 L265P	Wild-type	9	26.5
	Mutated	25	73.5
Treatment	With CD20 antibody	23	31.9
	Without CD20 antibody	49	68.1

**Figure 1:**
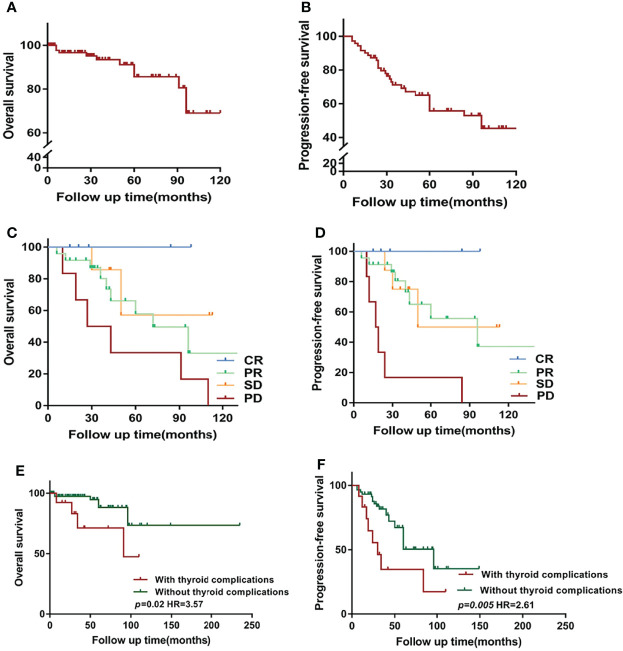
Overview of survival in Waldenström macroglobulinemia/lymphoplasmacytoid lymphoma (WM/LPL) patients. **(A)** Kaplan–Meier curves of overall survival (OS) in all enrolled WM/LPL patients. **(B)** Kaplan–Meier curves of progression-free survival (PFS) in all enrolled WM/LPL patients. **(C)** Comparison of OS by the response to initial treatment. **(D)** Comparison of PFS by the response to initial treatment. **(E)** Kaplan–Meier curves of OS stratified by thyroid complications. **(F)** Kaplan–Meier curves of PFS stratified by thyroid complications. CR, complete response; PR, partial response; SD, stable disease; PD, progressive disease.

Over a median of 38 months of follow-up, there was lost-to-follow-up in 38 cases (37%), death in 8 cases (7%), and progression developed in 47 cases (45%). The median OS of all patients was not reached (**Figure 1A**) and the median PFS was 96 months (**Figure 1B**). Furthermore, patients with no progression, including complete response (CR), partial response (PR), and stable disease, showed better OS (**Figure 1C**) and PFS (**Figure 1D**) than patients with progressive disease.

### Relationship Between Thyroid Function Abnormalities and Serum IgM Level in WM/LPL

In our study, a high incidence rate of thyroid complications was observed in WM/LPL patients. Given the abnormal IgM level as the most characteristic feature identified in WM/LPL patients’ sera, we wonder whether it might be related to thyroid complications. To investigate the potential association, we analyzed the correlation between thyroid complications and serum IgM levels. A relevant study showed that relative viscosity increased exponentially above a concentration of 30 g/L (3,000 mg/dl) (35). Moreover, it is reported that patients with IgM > 30 g/L are more likely to get symptomatic hyperviscosity (36). Thus, the patients were divided into low- and high-IgM groups with a cutoff of 30 g/L. Intriguingly, as **Table 2** shows, a significant correlation between thyroid complications and relatively alleviated IgM levels was observed (*p* = 0.03). However, there was no statistically significant correlation between the thyroid function parameters and IgM level, including FT3 (*p* = 0.246), FT4 (*p* = 0.6), Anti-TG (*p* = 0.576), and TSH (*p* = 0.128). These results suggested the potential causal associations between thyroid complications and alleviated IgM levels, providing an answer for clinical problems. However, further studies with larger sample sizes were required to validate the relationship between the level of thyroid hormones and IgM.

**Table 2:** Relationship between thyroid complications and serum immunoglobulin M (IgM) level in WM/LPL.

Characteristics	No. of patients	IgM level		*p*-value
		Low (≤30 g/L)	High (>30 g/L)	
FT3				
<Lower limit of normal	5	5	0	0.246
Normal	12	7	5	
FT4				0.600
<Lower limit of normal	5	3	2	
Normal	12	9	3
TSH				0.128
Normal	12	7	5	
≥Upper limit of normal	5	5	0
Anti-TG				0.576
Normal	10	7	3	
≥Upper limit of normal	2	1	1
Thyroid complications				
Absent	69	36	33	0.035*
Present	13	11	2	

*p < 0.05; Bold values mean statistically significant.

### Clinical Characteristics Correlated With Thyroid Function and IgM in WM/LPL

To evaluate the effect of thyroid function abnormalities and IgM on disease presentation, we examined the clinical and biological baseline information of the enrolled patients. The demographic and clinical features of the enrolled patients are depicted in **Table 3**. For clinical parameters, a significant dominance was identified in that patients without thyroid complications were more likely to get an overall response (CR+PR) to the initial treatment (*p* = 0.001). However, as to biological variables and laboratory findings, there were no indicators that presented statistical significance between WM/LPL patients with or without thyroid complications.

**Table 3:** Comparative clinical and biological characteristics of WM/LPL with or without thyroid complications.

Characteristics	No. of patients	Thyroid complications			*p*-value
		Absent	Present (*n* = 13)		
Age (years)					
<65	59	52		7	0.559
≥65	47	41		6	
Gender					
Male	72	65		7	0.197
Female	34	28		6	
Hemoglobin level (g/dl)					
≤11.5	58	50		8	0.525
>11.5	34	30		4	
Platelet (×10^9^/L)					
≤100	20	18		2	0.490
>100	72	62		10	
ISSWM risk group					
Low or intermediate (0–2)	45	42		3	0.014*
High (3–5)	28	20		8	
B-symptoms					
Absent	36	30		6	0.381
Present	31	24		7	
Extramedullary involvement					
Absent	64	60		4	0.211
Present	11	9		2	
MYD88 L265P mutation					
Negative	13	11		2	0.407
Positive	26	24		2	
Lactate dehydrogenase					
Normal	51	45		6	0.638
Elevated	11	10		1	
β2-microglobulin					
Normal	33	28		5	0.555
Elevated	48	40		8	
Globulin					
Normal	39	31		8	0.156
Elevated	48	43		5	
C-reactive protein Normal	30	25		5	0.258
Elevated	27	25		2	
Treatment					
Without CD20 antibody	27	21		6	0.503
With CD20 antibody	23	17		6	
Treatment response					
Response	32	30		2	0.001**
Non-response	15	7		8	

*p < 0.05, **p < 0.01; Bold values mean statistically significant.

Moreover, patients with high levels of IgM more frequently presented with high levels of globulin (*p* = 0.001), elevated β2-MG (*p* = 0.033), and decreased hemoglobin with *p*-values around 0.001. There were no differences related to sex, age, presence of B-symptoms, extramedullary involvement, MYD88 L265P mutation, and response to treatment (**Table 4**).

**Table 4:** Comparative clinical and biological characteristics of WM/LPL based on IgM level.

Characteristics	No. of patients	IgM level		*p-*value
		Low (≤30 g/L)	High (>30 g/L)	
Age (years)				
<65	48	30	18	0.383
≥65	36	19	17	
Gender				
Male	58	32	26	0.475
Female	26	17	9	
Hemoglobin (g/dl)				
<11.5	53	23	30	0.001***
≥11.5	30	25	5	
Platelet (×10^9^/L)				
<100	18	10	8	0.794
≥100	66	39	27	
ISSWM risk group				
Low or Intermediate (0–2)	43	24	19	0.326
High (3–5)	26	11	15	
B-symptoms				
Absent	54	32	22	0.817
Present	30	17	13	
Extramedullary involvement				
Absent	63	33	30	0.999
Present	21	11	10	
MYD88 L265P mutation				
Positive	26	9	17	0.995
Negative	55	19	36	
Lactate dehydrogenase				
Normal	50	26	24	0.121
Elevated	8	7	1	
β2-microglobulin				
Normal	31	24	7	0.033*
Elevated	48	25	23	
Globulin				
Normal	21	20	1	0.001***
Elevated	59	20	39	
Treatment response				
Response	30	12	18	0.226
Non-response	15	9	6	

*p < 0.05, ***p < 0.001; Bold values mean statistically significant.

### Prognostic Significance of Thyroid Complications and IgM in WM/LPL

The present study further investigated the prognostic value of thyroid complications and IgM in patients with WM/LPL. Kaplan–Meier curves showed no difference in OS between different groups of IgM levels (median follow-up time: low-IgM group, 40 months vs. high-IgM group, 42 months). A similar result was observed in PFS, with a *p*-value of 0.93. As demonstrated in **Figures 1E, F**, the median OS for patients with thyroid function abnormalities was significantly shorter than those without thyroid function abnormalities (not reached vs. 91 months, *p* = 0.02). Consistently, the result also indicated a PFS of 30 months for patients with thyroid complications, while the median survival was 60 months for non-thyroid complications (*p* = 0.005).

Subsequently, clinical and laboratory variables including age, ISSWM risk group, presence of thyroid complications, levels of hemoglobin, platelet, lactate dehydrogenase (LDH), globulin, and β2-MG, together with thyroid function parameters and cytogenetic parameters of MYD88 L265P mutated status, were entered into the univariate and multivariate Cox regression analyses. In the univariate Cox regression model, age (HR 0.403, 95% CI 0.185–0.879; *p* = 0.022), ISSWM score (HR 1.059, 95% CI 0.409–2.741; *p* = 0.014), and thyroid complications (HR 0.229, 95% CI 0.106–0.491; *p* < 0.001) were significantly associated with an increased risk of progression. The multivariate Cox analysis showed that the impact of thyroid complications was maintained irrespective of age and ISSWM score, with an HR of 0.226 for thyroid complications (95% CI 0.084–0.608, *p* = 0.03, **Table 5**).

**Table 5:** Univariate and multivariate Cox regression analyses of progression-free survival (PFS) in WM/LPL patients.

Variables		Univariate analyses				Multivariate analyses		
		HR	95% CI	*p-*value		HR	95% CI	*p-*value
Age (years)	<65 vs. ≥65	0.403	0.185–0.879		0.022*	0.223	0.043–1.149	0.073
ISSWM risk group	High vs. low/intermediate	1.059	0.409–2.741		0.014*	3.805	0.699–20.713	0.122
Immunoglobulin M	Low vs. high	1.219	0.513–2.899		0.654			
Hemoglobin	<115 vs. ≥115	1.461	0.586–3.641		0.416			
Platelet	<100 vs. ≥100	1.269	0.464–3.468		0.643			
LDH	<222 vs. ≥222	0.303	0.057–1.602		0.160			
β2-MG	<3 vs. ≥3	1.218	0.501–2.963		0.664			
Globulin	<35 vs. ≥35	0.642	0.192–2.137		0.326			
CRP	<10 vs. ≥10	1.043	0.408–2.669		0.930			
FT3	<3.1 vs. ≥3.1	0.947	0.252–3.554		0.935			
FT4	<12 vs. ≥12	1.007	0.201–5.032		0.993			
TSH	<4.2 vs. ≥4.2	4.254	0.258–4.254		0.948			
Thyroid complications	Absent vs. present	0.229	0.106–0.491		0.001***	0.226	0.084–0.608	0.03*
MYD88 L265P	Mutated vs. unmutated	0.498	0.130–1.913		0.310			

LDH, lactate dehydrogenase; β2-MG, β2-microglobulin; CRP, C-reactive protein; TSH, thyroid-stimulating hormone.

*p < 0.05, ***p < 0.001; Bold values mean statistically significant.

### Subgroup Analyses in Different Clinical Stages of Thyroid Dysfunction and ISSWM Score

**Figure 2:**
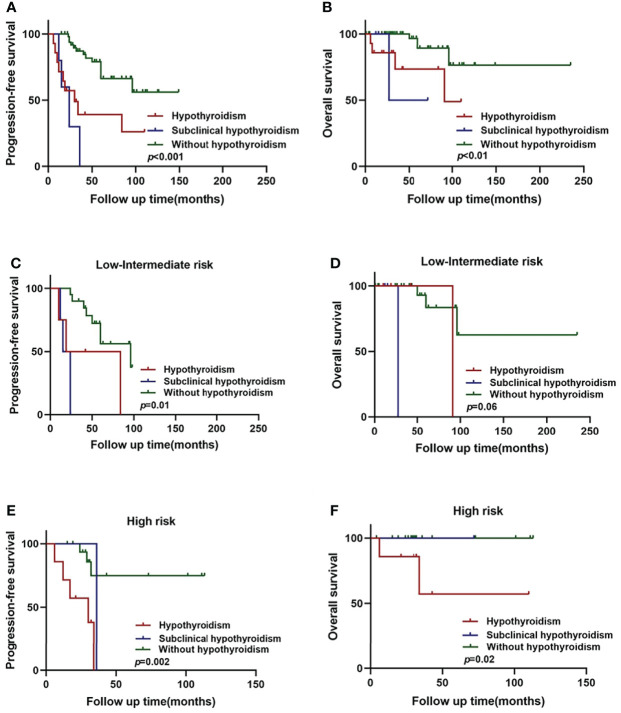
Hypothyroidism and subclinical hypothyroidism in relation to WM/LPL survival. **(A)** Kaplan–Meier curves of PFS stratified by hypothyroidism, subclinical hypothyroidism, and without hypothyroidism. **(B)** Kaplan–Meier curves of OS stratified by hypothyroidism, subclinical hypothyroidism, and without hypothyroidism. **(C)** Kaplan–Meier curves of PFS stratified by hypothyroidism, subclinical hypothyroidism, and without hypothyroidism in low-intermediate risk group. **(D)** Kaplan–Meier curves of OS stratified by hypothyroidism, subclinical hypothyroidism, and without hypothyroidism in low-intermediate risk group. **(E)** Kaplan–Meier curves of PFS stratified by hypothyroidism, subclinical hypothyroidism, and without hypothyroidism in high-risk group. **(F)** Kaplan–Meier curves of OS survival stratified by hypothyroidism, subclinical hypothyroidism, and without hypothyroidism in high-risk group.

Among the WM/LPL patients enrolled in our study, 8 cases were diagnosed as hypothyroidism, the most common of thyroid complications. The remaining 93 cases without thyroid complications were categorized into two subgroups: subclinical hypothyroidism and normal thyroid function parameters. The diagnosis of subclinical hypothyroidism was based on thyroid function testing: raised serum TSH concentrations and normal serum thyroid hormone concentrations (37). As shown in **Figures 2A, B**, a significant difference in PFS and OS was identified among the three subgroups, with the *p*-value around 0.001 in PFS and 0.01 in OS (log-rank test across all three groups).

As mentioned above, thyroid complications were significantly related to ISSWM score (*p* = 0.014). Therefore, we constructed the subgroup survival analysis for different ISSWM risk groups in WM/LPL patients. According to ISSWM, the entire cohort was divided into two risk grades: a low–intermediate-risk group (ISSWM 0-2) and a high-risk group (ISSWM 3-5). In the low–intermediate-risk group (**Figures 2C, D**), hypothyroidism and subclinical hypothyroidism patients were more likely to have an evidently shorter PFS (*p* = 0.01) compared with those patients without hypothyroidism, while no significant difference in OS (*p* = 0.06) was identified among the three subgroups. Subsequently, an evident survival difference was observed between patients with subclinical hypothyroidism and those without hypothyroidism in the early stage of WM/LPL. On the other hand, regardless of OS or PFS, there was no significance between the high-risk stage patients with subclinical hypothyroidism and those without hypothyroidism, while the high-risk stage patients with hypothyroidism were more likely to have evidently shorter PFS (*p* < 0.01) and OS (*p* < 0.05) (**Figures 2E, F**). These results indicate that hypothyroidism and subclinical hypothyroidism may have diverse outcomes at different stages of the patients, and WM/LPL patients with subclinical hypothyroidism should be given equal concern as those with hypothyroidism at the early ISSWM stage since they might share an undesirable survival outcome.

### Th-ISS, a Better Prognostic Index for WM/LPL

**Figure 3:**
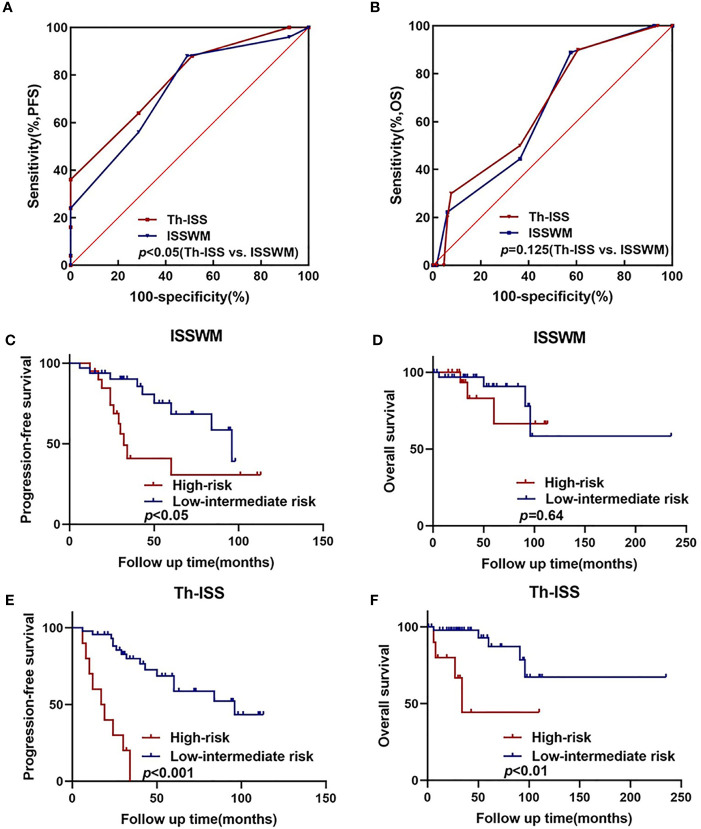
An extended ISSWM score based on thyroid complications. **(A, B)** The areas under the curve (AUC) comparison between prognostic index including thyroid complications alone, ISSWM, and combination (ISSWM) for PFS and OS prediction. **(C, D)** Kaplan–Meier curves of PFS and OS for different ISSWM risk grades. **(E, F)** Kaplan–Meier curves of PFS and OS for different Th-ISS risk grades.

Multivariate analysis showed that thyroid complications were the prognostic factor that independently predicted worse PFS in WM/LPL patients. Thus, adding the criterion of thyroid complications to ISSWM might improve its predictive capacity. Accordingly, a novel prognostic index (Th-ISS) can be generated by the sum of adding one point for thyroid complications to the original ISSWM. Considering the negative association between IgM levels and thyroid complications, thyroid complications concurrent with IgM ≤ 30 g/L will add two points to the original ISSWM. As a result, the Th-ISS index demonstrated a statistically significantly larger AUC compared with ISSWM alone in PFS evaluation (Th-ISS, AUC: 0.779, SE: 0.055, 95% CI: 0.667–0.867; ISSWM, AUC: 0.736, SE: 0.060, 95% CI: 0.620–0.831; *p* = 0.04) (**Figure 3A**). However, there is no evident significance in OS evaluation (Th-ISS, AUC: 0.733, SE: 0.091, 95% CI: 0.618–0.829; ISSWM, AUC: 0.652, SE: 0.087, 95% CI: 0.534–0.759; *p* = 0.12) (**Figure 3B**).

To further validate and confirm the prognostic capacity of Th-ISS, we firstly divided the cohort into two risk groups based on the original ISSWM: a low-intermediate risk group (ISSWM 0-2) and a high-risk group (ISSWM 3-5) (**Figures 3C, 3D**). However, only the PFS differed significantly among all risk groups according to ISSWM. For comparison, we next split the entire cohort into different risk grades based on the Th-ISS: a low-intermediate risk group (Th-ISS 0-3) and a high-risk group (Th-ISS 4-7). The PFS and OS differed significantly among all risk groups (p<0.001 and p<0.01, respectively) (**Figures 3E, F**). In pairwise comparison, an evident difference between OS and PFS was found between every pair of subgroups categorized by Th-ISS. Patients with high risk (Th-ISS 4-7) had worse survival. Thus, in conclusion, thyroid complications together with ISSWM can significantly differentiate each risk group from one another and improve the risk stratification of ISSWM.

### Analyses of Thyroid-Related Genes in WM/LPL Patients

**Figure 4:**
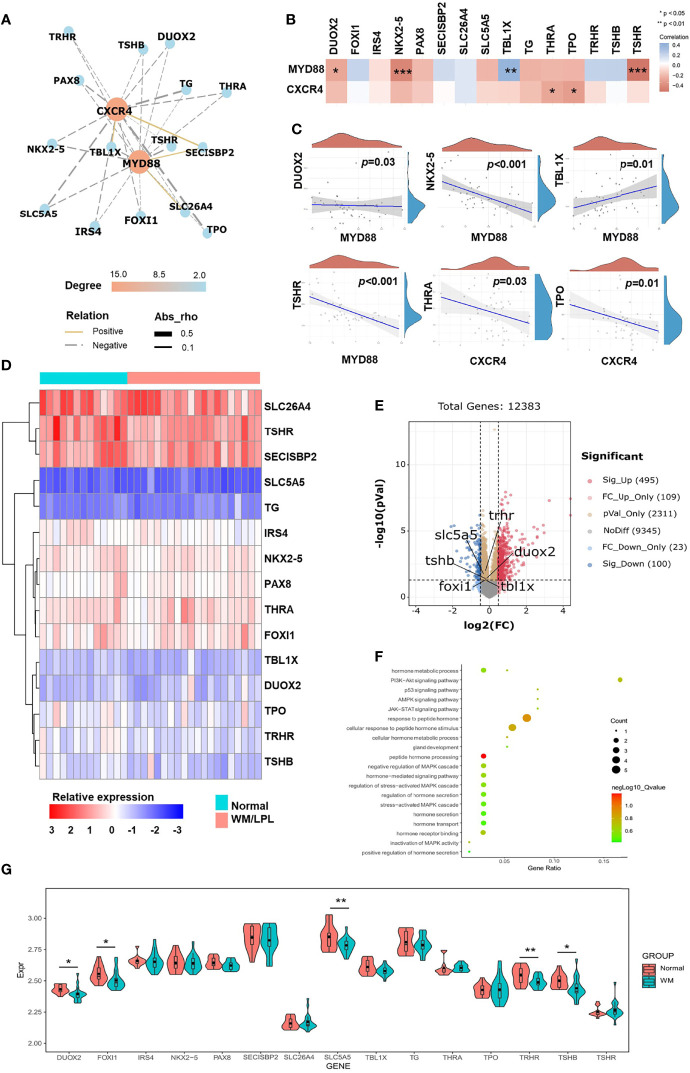
Correlation analyses and differential gene expression analyses between the WM/LPL and healthy donors. **(A)** Network diagrams of correlation for MYD88 and CXCR4 with thyroid-related genes in the WM/LPL patients. **(B)** A heat map of the correlation between MYD88, CXCR4, and thyroid-related genes. **(C)** Spearman correlation analysis of MYD88 and CXCR4 gene expression and thyroid-related genes (DUOX2, NKX2-5, TBL1X, TSHR, THRA, and TPO) expression. **(D)** Hierarchical clustering analysis of thyroid-related mRNAs between WM/LPL and healthy donors. **(E)** Volcano plots were constructed using fold-change values and adjusted *p*. The red point in the plot represents the over-expressed mRNAs and the blue point indicates the under-expressed mRNAs with statistical significance. **(F)** The enriched KEGG signaling pathways and GO analysis were selected to demonstrate the primary biological actions of thyroid-related mRNA. **(G)** The expression distribution of thyroid-related mRNA in WM/LPL and control groups. Asterisks represent levels of significance (*p < 0.05, **p < 0.01, ***p < 0.001).

The above findings prompted us to further investigate the role of thyroid-related genes in WM/LPL patients. To make it easier to demonstrate the interplay between thyroid-related genes and critical factors in WM/LPL, including MYD88 and CXCR4, we constructed a network of interactions among the genes (**Figure 4A**). After that, the significance of the interaction was assessed by the Pearson correlation coefficient. As shown in **Figure 4B**, the ordinate in the figure represents the expression distribution of MYD88 and CXCR4, and the horizontal axis represents the expression distribution of thyroid-related genes. Different colors represent correlation coefficients (in the diagram, red represents positive correlation and blue represents negative correlation). Color intensity indicates statistical significance. Statistically significant correlations between MYD88, CXCR4, and thyroid-related genes are shown in **Figure 4C**.

Subsequently, differential gene expression analysis between the WM/LPL and healthy donors was assessed. As shown in **Figure 4D**, a hierarchical cluster analysis was performed on differentially expressed genes between WM/LPL patients and healthy donors. We then constructed volcano plots using fold-change values and adjusted *p*. The red point in the plot represents the over-expressed mRNAs and the blue point indicates the under-expressed mRNAs with statistical significance. Thyroid-related genes with statistical significance were annotated in the graph (**Figure 4E**). Functional enrichment analysis of thyroid-related gene expression in WM/LPL microarray profiles was performed. Gene ontology (GO) analysis and Kyoto Encyclopedia of Genes and Genomes (KEGG) enrichment analysis indicated that thyroid-related gene was involved in response to peptide hormone and the PI3K-Akt signaling pathway (**Figure 4F**). The expression of thyroid-related genes between WM/LPL patients and healthy donors was shown using a box-violin plot. Five of them reached statistical significance, and one (TBL1X) reached marginal significance (**Figure 4G**).

## Discussion

Thyroid dysfunction has been found to be associated with prognosis in a wide array of morbidities, including cardiovascular disease, chronic kidney disease, and several forms of cancer. To our knowledge, this is the first study that provides evidence of thyroid complications as a strong indicator of poor prognosis among patients with WM/LPL. WM/LPL patients with thyroid complications showed undesirable treatment responses, which suggested a more aggressive therapeutic approach for these patients. An extended prognostic system of ISSWM based on thyroid complications was established and improved the risk stratification of ISSWM. In addition, significant correlations between thyroid complications and unfavorable clinical characteristics were identified, highlighting the necessity of closer monitoring of WM/LPL patients with thyroid function abnormalities.

In the last two decades, thyroid dysfunction has been reported to be highly prevalent in critically ill patients, including respiratory failure, severe trauma, end-stage renal disease, and acute stroke (38–42). For the presence of thyroid dysfunction in malignancies, it was reported that low T3 syndrome was common in patients with brain tumors and lung cancer (43–45). Patients with plasma cell dyscrasias seem to be at an increased risk of thyroid cancer (46). We also found a high incidence (13.3%) of thyroid complications among 106 WM/LPL patients enrolled in our study, which ranks as the third most common complication. Trimarchi et al. reported an abnormal binding of thyroid hormones in the gamma-globulin fraction in a 68-year-old patient with WM (47). Consistently, we found a significant correlation between thyroid function abnormalities and alleviated serum IgM levels. The correlation between thyroid-related genes and WM/LPL was further supported by bioinformatics results. Thus, we speculate that the reduced IgM might arise from the abnormal binding of thyroid hormones and IgM. High IgM levels are one of the most striking features of patients with WM, but the serum IgM levels may vary when thyroid disease is present. However, the relationship between thyroid function parameters and IgM remains unclear due to the limited available data. Although the pathophysiological mechanism was ambiguous, the negative association between thyroid complications and immunoglobulins still suggested that immunoglobulin treatment should be used with caution for those with thyroid complications.

The associations among thyroid dysfunction, inflammatory state, malnutrition, and anemia were depicted by Fan et al., suggesting the positive correlation between thyroid dysfunction and anemia (hemoglobin) as well as protein-energy nutrition (serum albumin), and the negative correlation between serum T3 and inflammation (C-reactive protein) (48). In agreement with these findings, serum IgM was closely correlated with anemia parameters and LDH. β2-MG was observed to be involved in the presence of thyroid complications in this study, suggesting an abnormal inflammatory state in WM/LPL patients with thyroid complications.

Prior epidemiological studies have shown that thyroid hormone dysfunction is an independent prognostic factor for poor survival in patients with severe burns, sepsis, acute cerebrovascular disease, chronic heart failure, end-stage renal disease, and hemodialysis (49–52). However, the role of thyroid complications in cancer prognosis has only been well-analyzed in glioma and lung cancer as a predictor of unfavorable clinical prognosis. As an independent risk factor for PFS in WM/LPL patients, thyroid complications were demonstrated in our study. Based on these findings, the thyroid complications were then combined with the ISSWM score as the extended prognosis score, which showed a better risk stratification. Our findings suggested that hypothyroidism was associated with worse PFS and OS in WM/LPL patients, especially those patients at high-risk stage, and subclinical hypothyroidism in early-stage patients should be given more attention. It was also observed that patients with thyroid complications had the worst treatment response. Since the biological mechanism of this association is not yet clear, thyroid complications can be interpreted as a risk factor for increased mortality, rather than a direct cause of poor prognosis in WM/LPL. Intriguingly, the prognostic value of IgM was not observed in either treatment response or survival. The same result was reported in a nationwide prospective Swedish registry-based study of WM (53). However, incorporating thyroid complications and IgM ≤ 30 g/L into the same prognostic system significantly improved the prediction performance. This interesting observation could explain why some patients with relatively low IgM levels refer to unfavorable survival. Relevant studies have shown that the thyroid is more susceptible to autoimmunity than other organs; thus, thyroid complications have been considered to be manifestations of autoimmunity problems (27). IgM ≤ 30 g/L co-existing with thyroid complications indicates an abnormal immune state in WM/LPL patients.

Previous studies have reported the potential correlation between anti-CD20 treatment and thyroid complications in B-cell lymphoma. Thyroid dysfunction has been identified as the third most common low-grade adverse event, accounting for 27% in a clinical trial of pembrolizumab in combination with rituximab in follicular lymphoma (54). Another case of thyroid diffuse large B-cell lymphoma was diagnosed as Graves’ disease following R-CHOP treatment (55). Along with this observation, one person was also diagnosed with Graves’ disease following anti-CD20 treatment in our data. However, it accounted for 4.3% of the 23 WM/LPL patients after anti-CD20 treatment. The association between thyroid complications and the anti-CD20 regimen was not significant. Thus, based on the current evidence, it is unclear whether the thyroid complications originate from anti-CD20 treatment. Further large-sample multicenter studies with a statistical perspective are suggested to demonstrate the potential link between thyroid complications and anti-CD20 treatment.

In addition, there were still several limitations in our study. This investigation was limited by the cross-sectional research design; a prospective design collecting data on the same participants would likely yield results more specific to the effect of thyroid complications. An additional limitation of the study was the small sample size within one institution. There could be unknown imbalances between with and without thyroid complication cohorts that could cause bias. Finally, the pathophysiologic correlation between thyroid complications and monoclonal immunoglobulin is not fully elucidated, which requires further experiments to investigate the underlying mechanisms.

In conclusion, thyroid complications were significantly correlated with serum IgM levels in WM/LPL patients. Those patients showed worse clinical characteristics, and unfavorable PFS and OS. Furthermore, the presence of thyroid complications retained independent prognostic significance for PFS. Extended prognostic index with thyroid complications as an additional point to ISSWM showed improved predictive performance and risk stratification. Future longitudinal studies with a longer follow-up and larger sample sizes are warranted to verify the role of thyroid hormones in a WM/LPL population.

## Data Availability Statement

The original contributions presented in the study are included in the article/supplementary material. Further inquiries can be directed to the corresponding authors.

## Ethics Statement

The studies involving human participants were reviewed and approved by the Research Ethics Committee of the Shandong Provincial Hospital. The patients/participants provided their written informed consent to participate in this study.

## Author Contributions

XW and YZ conceived the paper and edited the manuscript. XH and HW analyzed the data and wrote the first draft of the manuscript. HX, XL, and YL collected the data. XHW, NW, DY, and HQ performed the statistical analyses. All authors contributed to the article and approved the submitted version.

## Funding

This study was funded by the National Natural Science Foundation (No. 82000195, No. 82070203, and No.81770210), the Key Research and Development Program of Shandong Province (No. 2018CXGC1213), the Translational Research Grant of NCRCH (No. 2021WWB02 and No. 2020ZKMB01), the Taishan Scholars Program of Shandong Province, the Shandong Provincial Natural Science Foundation (No. ZR2020QH094), the Shandong Provincial Engineering Research Center of Lymphoma, the Technology Development Project of Jinan City (No. 202019182), the Academic Promotion Programme of Shandong First Medical University (No. 2019QL018 and No. 2020RC007), and the Shandong Provincial Hospital Youth Talent Plan.

## Conflict of Interest

The authors declare that the research was conducted in the absence of any commercial or financial relationships that could be construed as a potential conflict of interest.

## Publisher’s Note

All claims expressed in this article are solely those of the authors and do not necessarily represent those of their affiliated organizations, or those of the publisher, the editors and the reviewers. Any product that may be evaluated in this article, or claim that may be made by its manufacturer, is not guaranteed or endorsed by the publisher.
